# Mitochondrial Genomes of Six *Discogobio* Species (Teleostei, Cyprinidae) and Their Phylogenetic Analysis

**DOI:** 10.1002/ece3.71142

**Published:** 2025-03-17

**Authors:** Huan Cheng, Renrong Huang, Hongmei Li, Zhenya Qiu, Shan Xiong, Renyi Zhang

**Affiliations:** ^1^ School of Life Sciences Guizhou Normal University Guiyang Guizhou China

**Keywords:** divergence time, Labeoninae, mitogenome, phylogeny, selection pressure

## Abstract

*Discogobio* is an important small freshwater economic fish in Southwestern China. In this study, we determined the complete mitochondrial genome (mitogenome) sequences of six *Discogobio* species by conventional overlapping PCR and Sanger sequencing. The mitogenomes were 16,591–16,605 bp in length and contained 13 protein‐coding genes, 22 transfer RNA genes, two ribosomal RNA genes, and a noncoding region, which was consistent with previously studied Labeoninae mitogenomes. The phylogenetic analysis revealed that the subfamily Labeoninae was divided into four major clades. Notably, *Discogobio* was observed to be a non‐monophyletic group and mixed with *Discocheilus*. The divergence time of *Discogobio* and *Discocheilus* can be placed during the time of the most recent common ancestor in the late Miocene (7.80 Mya). The results of the selection pressure analyses indicated that all *Discogobio* fishes exhibited Ka/Ks ratios < 1, suggesting that mitochondrial function in this genus was subjected to strong purifying selection and adapted to different environments. These mitogenomes will facilitate further studies in phylogeny, taxonomy, and evolutionary biology related to the subfamily Labeoninae.

## Graphical abstract

In this study, we sequenced and analyzed the complete mitochondrial genome sequences of six *Discogobio* species by conventional overlapping PCR and Sanger sequencing. Additionally, we investigated the phylogenetic history of the *Discogobio* genus.

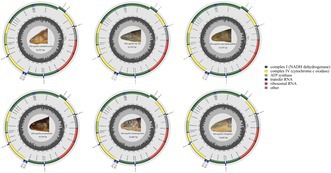

## 1 Introduction

The mitogenome of fish is a circular, double‐stranded DNA molecule ranging from 15 to 20 kb in size (Brown 2008; Zhang, Zhou, et al. 2023; Zhang, Zhu, et al. 2023). It generally contains 37 genes, which include 13 protein‐coding genes (PCGs), 22 transfer RNA genes (tRNAs), and two ribosomal RNA genes (rRNAs), as well as one noncoding region known as the control region (Brown 2008; Zhang, Zhou, et al. 2023; Zhang, Zhu, et al. 2023). The mitogenome offers several advantages, including its relatively small size, double circular structure, simple architecture, rapid evolutionary rate, and low recombination levels (Avise et al. 1984; Moritz et al. 1987; Xiao and Zhang 2000). As a consequence of these characteristics, it constitutes an invaluable instrument for studying molecular evolution and phylogenetic relationships (Shen et al. 2020; Yang et al. 2021).

The genus *Discogobio* is classified within the order Cypriniformes, family Cyprinidae, and subfamily Labeoninae. These fishes are distinguished by their unique mouth suckers, which exhibit a prominent horseshoe‐shaped fold in the center, a characteristic that sets them apart within the subfamily (Zhang et al. 2000). *Discogobio* species are small, benthic fishes that have evolved to thrive in fast‐flowing environments. They are typically found in the main and tributary streams of upper rivers, as well as in streams and lakes. The distribution of this genus is currently limited to China and Vietnam, with a significant concentration in the Pearl River, Nanpanjiang River, and Yuanjiang River systems in Southwestern China. According to Eschmeyer's Catalog of Fishes, the genus currently comprises 16 valid species (Fricke et al. 2024).

Molecular phylogenetic studies have clarified the phylogenetic relationships within the subfamily Labeoninae and confirmed the classification of its genera (Yang and Mayden 2010; Zheng et al. 2010, 2012; Yang et al. 2012). The subfamily Labeoninae was divided into four major clades (Yang et al. 2012), with the karst group identified as part of the fourth clade (Zheng et al. 2016). Additionally, the genus *Discogobio* was also found to belong to the karst group. Previous studies also indicated that this genus was non‐monophyletic and closely related to *Discocheilus* (Zheng et al. 2010; Zheng and Geng 2024). Only some species have mitochondrial genome data, which has left the molecular phylogeny, evolutionary history, and interrelationships within *Discogobio* insufficiently understood.

This study expands the existing data on mitogenomes for the genus *Discogobio* by analyzing and describing six complete mitogenomes. We reconstructed the phylogenetic tree of *Discogobio* using all available mitogenomes from GenBank to gain a deeper understanding of the phylogeny, divergence time, and adaptive evolution of this group. The findings of this study will undoubtedly provide valuable insights for future research in population genetics and the conservation biology of these species.

## 2 Materials and Methods

### 2.1 Sample Collection

**FIGURE 1:**
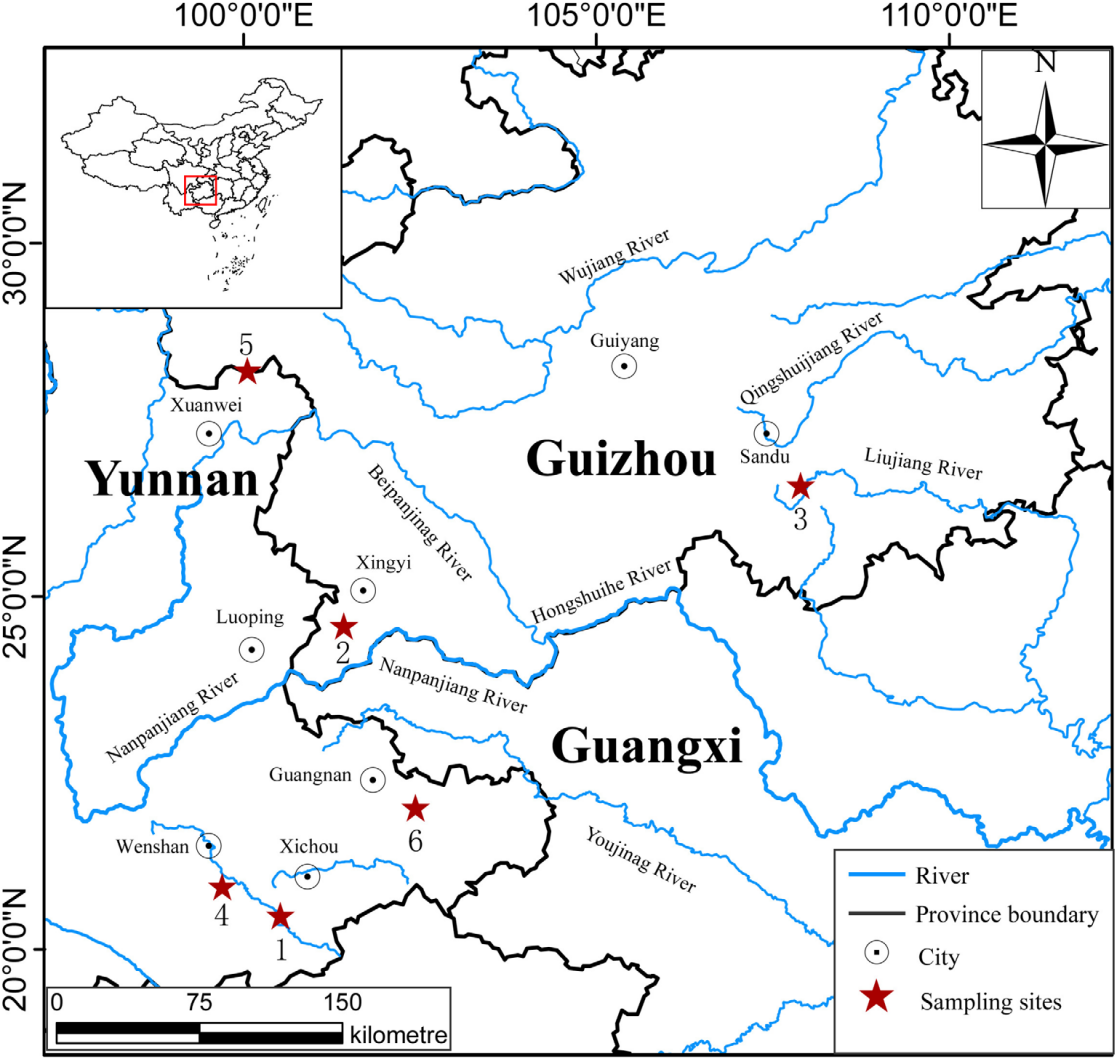
Map of six species sampling sites. Codes for locations can be found in Table 1.

Six species of *Discogobio* (*D. antethoracalis* (DA), *Discogobio* sp. SZ (SZ), *D. macrophysallidos* (DM), *D. propeanalis* (DP), *D. bismargaritus* (DB), and *D. elongatus* (DE)) were collected from Guizhou and Yunnan provinces in China between 2022 and 2024 (Figure 1; Table 1). The samples of DB and DE were kindly provided by Hongfu Yang, while the remaining samples were purchased from local fishermen. These specimens were then preserved in 75% alcohol as voucher specimens and deposited at the School of Life Sciences, Guizhou Normal University.

**TABLE 1:** Sampling details for six species used in this study.

Code	Species	Location	River drainage	Collection date
1	*Discogobio antethoracalis*	Xichou County, Yunnan Province, China (23°09′ N, 104°27′ E)	Panlonghe River	August 14, 2024
2	*Discogobio* sp. SZ	Sandu County, Guizhou Province, China (25°54′ N, 107°44′ E)	Duliujiang River	July 31, 2022
3	*Discogobio macrophysallidos*	Xingyi City, Guizhou Province, China (25°01′ N, 104°52′ E)	Nanpanjiang River	July 22, 2022
4	*Discogobio propeanalis*	Wenshan City, Yunnan Province, China (23°22′ N, 104°06′ E)	Shundianhe River	August 14, 2024
5	*Discogobio bismargaritus*	Guangnan County, Yunnan Province, China (23°52′ N, 105°19′ E)	Xiyangjiang River	July 16, 2024
6	*Discogobio elongatus*	Xuanwei City, Yunnan Province, China (26°37′ N, 104°15′ E)	Beipanjiang River	April 13, 2024

### 2.2 DNA Extraction, PCR Amplification, and Sequencing

The total genomic DNA was extracted from the muscle tissue samples using the standard high‐salt digestion method (Aljanabi and Martinez 1997). The extracted genomic DNA was employed as the template for polymerase chain reaction (PCR) amplification. Based on the complete mitochondrial genome sequence of *D. yunnanensis* (GenBank accession number: NC025319), a total of 15 primers were designed using Primer Premier 5.0 (https://www.premierbiosoft.com/primerdesign/) (Table S1). The polymerase chain reaction (PCR) was conducted in a total volume of 35 μL, comprising 17.5 μL of 2x Taq Plus Master Mix (Cwbio, Beijing, China), 14.5 μL of ddH_2_O, 1 μL of template DNA (100 ng/μL), and 1 μL of each primer (10 μM/L).

The PCR conditions were as follows: an initial denaturation at 95°C for 5 min, followed by 35 cycles of denaturation at 95°C for 1 min, annealing at 45°C–56°C (Table S1) for 30 s, extension at 72°C for 1–1.5 min, and a final extension at 72°C for 10 min. The amplification of the target sequence was confirmed by observing the PCR products on a 1% agarose gel. The size of the PCR products was determined by comparison with the DL2000 DNA size marker. The target PCR products were then sequenced in both directions using an ABI 3730 sequencer (Sangon Biotech, China).

### 2.3 Assembly, Annotation, and Sequence Analysis

The sequence fragments were assembled using SeqMan software (DNA STAR package; DNAStar Inc., Madison, WI, USA). Once assembled, the mitogenome sequences were automatically annotated using MitoAnnotator (https://mitofish.aori.u‐tokyo.ac.jp/annotation/input/) (Iwasaki et al. 2013). tRNA genes were further identified and annotated through the MITOS Web Server and the tRNAscan‐SE2.0 search server (http://lowelab.ucsc.edu/tRNAscan‐SE/) (Bernt et al. 2013; Lowe and Chan 2016). The base composition and codon usage were calculated using MEGA v7.0 software (Kumar et al. 2016). The relative synonymous codon usage (RSCU) for each PCG was analyzed with PhyloSuite v1.2.3 (Zhang et al. 2020). To further analyze the sequences, A + T skew and G + C skew were determined using standard formulas: the A + T skew was defined as (A% − T%) / (A% + T%), while the G + C skew was defined as (G% − C%) / (G% + C%) (Perna and Kocher 1995).

### 2.4 Phylogenetic Analyses

The phylogenetic relationships were reconstructed using the PCGs from 110 mitogenomes, including the six newly sequenced species and two outgroups ( *Zacco platypus* and *Xenocypris macrolepis* ) (Table S2). The PCGs were extracted using PhyloSuite v1.2.3 (Zhang et al. 2020) and aligned using MAFFT v7.0 (Katoh and Standley 2013). Subsequently, phylogenetic analyses were conducted using maximum likelihood (ML) and Bayesian inference (BI) methods. The partitioning scheme and evolutionary models for the 26 predefined partitions were selected using the greedy algorithm and the corrected Akaikeinformation criterion (AICc) in PartitionFinder 2 v2.1.1 (Lanfear et al. 2017). The ML phylogenetic tree was constructed with IQ‐tree v1.6.12 (Nguyen et al. 2015), utilizing an edge‐linked partition model with 5000 ultrafast bootstraps (Minh et al. 2013) and the Shimodaira‐Hasegawa‐like approximate likelihood ratio test (Guindon et al. 2010). The BI analysis was conducted using MrBayes v3.2.6 (Ronquist et al. 2012), with two independent Markov Chain Monte Carlo (MCMC) analyses, each comprising four chains and a total of two million generations. The initial 25% of samples from each MCMC run were excluded as a result of the burn‐in phase. The resulting phylogenetic tree was visualized using the online tool “Interactive Tree Of Life” (iTOL) (https://iTOL.embl.de/, accessed 15 July 2024) (Letunic and Bork 2019).

### 2.5 Divergence Time and Evolutionary Rate Estimation

In order to estimate the divergence times among *Discogobio* species, a Bayesian analysis was conducted using the software BEAST v2.6.0 (Bouckaert et al. 2014). The BEAST XML file was created with BEAUTi 2, employing a strict clock model and a Yule speciation process as the tree prior. Given the paucity of fossil evidence pertaining to *Discogobio* fishes, we employed a node‐secondary calibration and fossil calibration approach, utilizing genome‐estimated time nodes of differentiation in *Discogobio* fishes for calibration purposes. (1) Derived from the oldest fossil record in Loperot, Kenya, dated to the early Miocene (~17 million years ago, Mya) (Van Couvering 1977; Stewart 2001). (2) Origin of the labeonin fishes. The second uplift of the Qinghai‐Xizang Plateau occurred between 25 and 17 million years ago (Shi et al. 1999). The divergence times were estimated using the GTR model, with a gamma distribution for rate variation among sites and the default model parameter priors. The initial 20% of cycles were excluded from the analysis as burn‐in, and sampling was performed every 1000 generations from a total of 50,000,000 Markov Chain Monte Carlo iterations. The tree was annotated using TreeAnnotator (part of the BEAST suite) and visualized using Figtree v1.4.3 (http://tree.bio.ed.ac.uk/software/figtree/). Subsequently, Chiplot was utilized for the generation of corresponding graphics (Xie et al. 2023). The Bayesian significance of each parameter was evaluated using the Effective Sample Size (ESS > 200) in TRACER v1.7.1 (Rambaut et al. 2018).

To assess the selection pressure on the *Discogobio* species, we employed the EasyCodeML v1.0 software (Gao et al. 2019), a component of the PAML package, to run a free‐ratio model (model = 1) on all 13 concatenated PCGs. This analysis employed a single model mode, enabling the estimation of independent Ka/Ks rates (nonsynonymous to synonymous substitution rates) for each branch of the tree (Sun et al. 2011).

## 3 Results and Discussion

### 3.1 Mitogenomes Structure, Organization, and Nucleotide Composition

**FIGURE 2:**
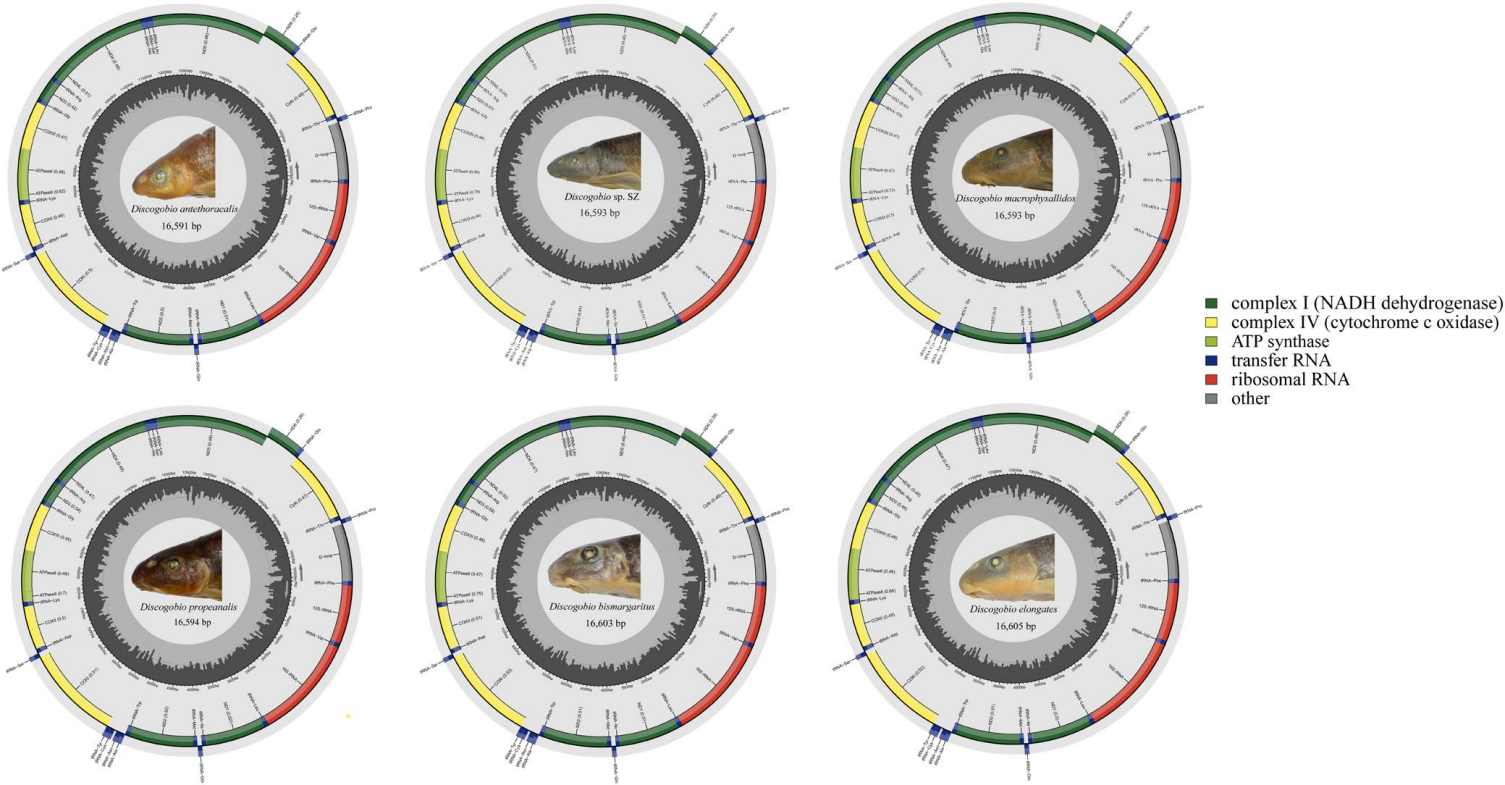
Gene maps of the six *Discogobio* mitogenomes.

In this study, we presented the complete mitogenomes of the six *Discogobio* with lengths of 16,591–16,6605 bp for DA, *Discogobio* sp. SZ, DM, DP, DB, and DE (GenBank accession numbers: PQ464574–PQ464575 and PQ789988–PQ789991). The mitogenome composition and structure of these six species were highly concordant. Each mitogenome contained 13 PCGs (COXI–XIII, Cytb, ND1–6, ND4L, ATP6, and ATP8), 22 tRNA genes, two RNA genes (12S rRNA and 16S rRNA), and one D‐loop (Figure 2; Table S3). Among these genes, 28 (12 PCGs, 14 tRNAs, and two rRNAs) were encoded on the heavy chain (H chain), while nine (one PCG and eight tRNAs) were encoded on the light chain (L chain) (Figure 2; Table S3). The arrangement and composition of the mitochondrial genes in these species were consistent with those found in most other Labeoninae species (Zhao et al. 2014; Zheng and Yang 2017a; Zhang, Zhou, et al. 2023; Zhang, Zhu, et al. 2023; Zheng and Geng 2024).

**FIGURE 3:**
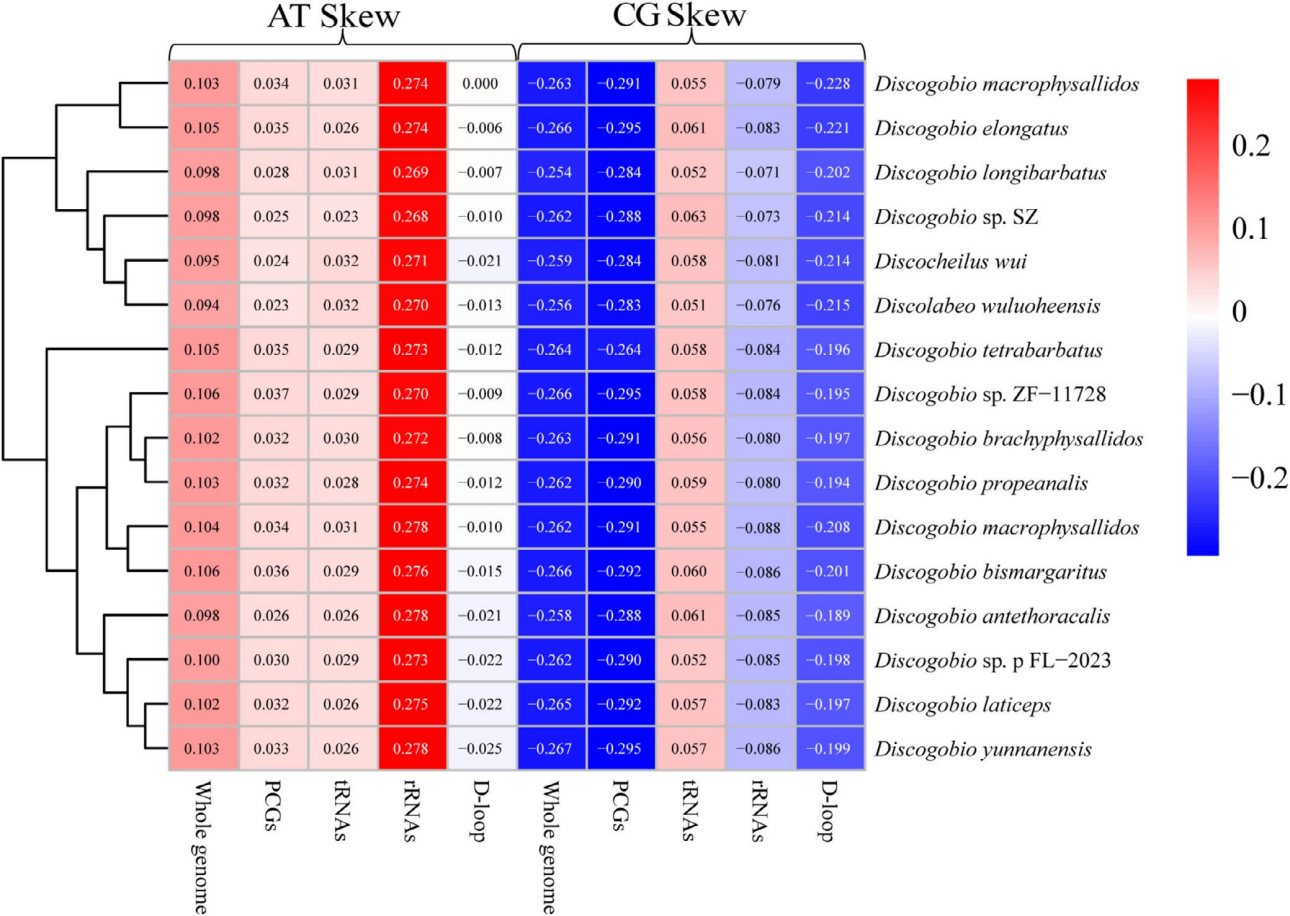
Nucleotide composition of various datasets of mitogenomes. Hierarchical clustering of 16 *Discogobio* and *Discolabeo* species (*y*‐axis) based on the AT‐skew and GC‐skew.

The overall A + T contents of DA, *Discogobio* sp. SZ, DM, DP, DB, and DE were similar (58.0%–58.7%) (Table S4). The six newly sequenced mitogenomes exhibit high A + T values, which were consistent with the typical base bias of fish mitogenomes (Zhang, Zhou, et al. 2023; Zhang, Zhu, et al. 2023; Zheng and Geng 2024). The nucleotide compositions of 16 mitogenomes in *Discogobio* and *Discocheilus* were investigated by calculating the percentages of AT‐skew and GC‐skew (Figure 3). The results of the nucleotide skew statistics showed that the AT‐skews in the whole genome, PCGs, tRNAs, and rRNAs of 16 species were all positive. In contrast, the GC‐skews were predominantly negative except for the tRNAs. The pattern of nucleotide skewness in the *Discogobio* mitogenomes was consistent with those of most other Labeoninae (Tan et al. 2019; Chen et al. 2024; Zheng and Geng 2024).

### 3.2 Characteristics of PCGs and Codon Usage

The six newly sequenced mitogenomes contained 13 PCGs, with ATP8 being the smallest (165 bp) and ND5 being the largest (1824 bp), which was consistent with other *Discogobio* mitogenomes (Zheng and Yang 2017b; Chen et al. 2024). The initiation codons for the COXI gene of six species were GTG, whereas all other PCGs consistently used the ATG initiation codon (Table S3). The COXI gene with GTG as the start codon is a common phenomenon in fish mitogenomes (Tan et al. 2019; Qiu et al. 2020; Zhang, Zhou, et al. 2023; Zhang, Zhu, et al. 2023). All 13 PCGs were terminated with TAA, TA, or T codons. Notably, ND6 in *Discogobio* sp. SZ and ATP8 in DB were terminated with TAG. Incomplete stop codons were a common feature of vertebrate mitogenomes and were believed to be completed through posttranscriptional modifications, such as polyadenylation (Ojala et al. 1981).

The amino acid content, composition, and codon usage of the 13 PCGs in these six species were relatively similar (Figure S1). Leu, Ala, Thr, and Ile were used relatively frequently, while Cys was the least common amino acid. When comparing the relative synonymous codon usage (RSCU) of the six species, it was found that the usage of codons GCU (Ala), CCU (Pro), UUA (Leu2), and ACU (Thr) was relatively frequent and higher than that of other codons.

### 3.3 Characteristics of rRNAs, tRNAs, and Control Region

The lengths of 16S rRNA genes ranged from 1684 to 1685 bp, whereas those of 12S rRNA were 953 bp (Table S3). The two rRNA genes were found to be located between tRNA^Phe^ and tRNA^Leu2^ and isolated by tRNA^Val^. The A + T contents of 16S rRNA and 12S rRNA range from 56.9% to 57.4% and 51.4% to 52.4%, respectively, which shows a slight AT bias (Table S4).

The lengths and locations of tRNA genes in the six species of *Discogobio* were generally consistent (Figure 2; Table S3). The lengths of the tRNA genes ranged from 67 bp (tRNA^Cys^) to 76 bp (tRNA^Leu2^ and tRNA^Lys^) (Table S3). All tRNA genes had standard anticodons and could be folded into the typical cloverleaf structure, except for tRNA^Ser1^, which lacked a dihydrouridine (DHU) arm in all six sequenced mitogenomes (Figures [Link], [Link], [Link], [Link], [Link], [Link]). The AT‐skew values ranged from 0.023 to 0.031, while the GC‐skew values varied from 0.055 to 0.063, indicating that the numbers of A and C were greater than those of T and G. The characteristics of these tRNA genes were consistent with those found in the mitochondrial DNA of metazoans (Watanabe et al. 2014).

The control region (D‐loop), located between tRNA^Pro^ and tRNA^Phe^, spans from 933 to 945 bp in six species (Figure 2; Table S3). This region contains the highest proportion of adenine and thymine, ranging from 67.7% to 69.2% (Table S4).

### 3.4 Phylogenetic Analyses

**FIGURE 4:**
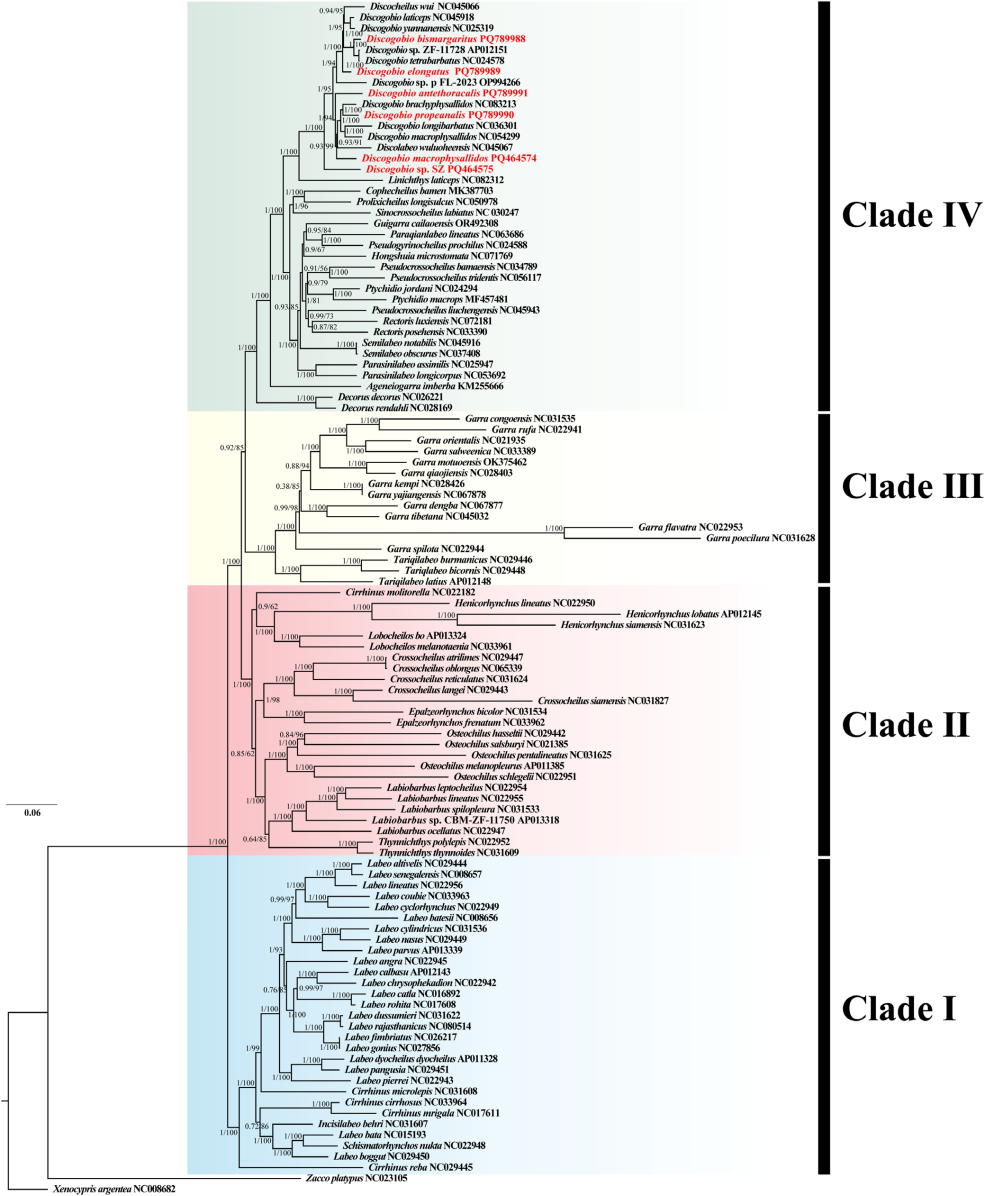
Phylogenetic tree inferred using Bayesian inference (BI) and maximum likelihood (ML) methods based on concatenated sequences of 13 PCGs from 110 mitogenomes. The numbers on each node indicate the posterior probability values from the BI analysis (left) and the ML bootstrap percentage values from 1000 replicates of the maximum likelihood analysis (right). The number following each species name represents the GenBank accession number.

Phylogenetic analyses were performed based on the concatenated nucleotide sequences of 13 PCGs from 108 species belonging to 34 families within the Labeoninae subfamily, with corresponding sequences from *Zacco platypus* (NC023105) and *Xenocypris macrolepis* (NC008682) as outgroups. Both maximum likelihood and Bayesian inference analyses yielded nearly identical topologies, with strong bootstrap and posterior probability values (Figure 4). The subfamily Labeoninae can be divided into four major clades, namely Clade I, Clade II, Clade III, and Clade IV (Figure 4). This classification was consistent with findings from previous research (Yang et al. 2012; Zheng and Geng 2024). Clade I was composed of eight genera: *Crossocheilus*, *Bangana*, *Incisilabeo*, *Labeo*, *Sinocrossocheilus*, *Pseudogyrinocheilus*, *Schismatorhynchos*, and *Squalius*. Clade II consists of seven genera: *Cirrhinus*, *Epalzeorhynchos*, *Osteochilus*, *Henicorhynchus*, *Lobocheilos*, *Labiobarbus*, and *Thynnichthys*. Clade III was made up of two genera: *Garra* and *Tariqilabeo*. Clade IV comprises sixteen genera, including *Ageneiogarra*, *Cophecheilus*, *Discocheilus*, *Discogobio*, *Decorus*, *Guigarra*, *Hongshuia*, *Linichthys*, *Paraqianlabeo*, *Prolixicheilus*, *Pseudocrossocheilus*, *Ptychidio*, *Parasinilabeo*, *Rectoris*, *Semilabeo*, and *Sinocrossocheilus*. *Discogobio* belongs to Clade IV and is not a monophyletic group, being intertwined with *Discocheilus*. Both *Discocheilus* and *Discogobio* have discs on the lower lips, although there are subtle differences in the discs and lips between them (Zheng et al. 2010; Zheng and Geng 2024). Zheng et al. (2010) conducted molecular studies which revealed that the minute differences in the rostral fold and disc alone were inadequate for defining two monophyletic genera. This suggests that there may be some problems with the classification of these two genera that need to be resolved.

Based on the results of morphological identification and phylogenetic analysis, SZ showed characteristics of an independent species within the genus *Discogobio*. Therefore, SZ was currently classified as an undetermined species. In this study, the DM sample was morphologically identified to be consistent with DM. However, phylogenetic analysis indicates that DM does not cluster with the previously recorded DM (NC054299). The type locality of DM was Fuyuan and Luoping in Yunnan Province, China, while the type locality of *D. polylepis* was Fuxian Lake, Yunnan Province, China (Huang 1989). Subsequently, *D. polylepis* was deemed invalid and considered a synonym of DM (Chu et al. 1993). However, Zheng (2007) reinstated the validity of *D. polylepis* after reviewing the type specimen and recognized it as a distinct species. Therefore, it was recommended that the sequence of DM (NC054299) uploaded by Qiu et al. (2020) be corrected to *D. polylepis* , which was sampled from Xingyun Lake.

### 3.5 Divergence Time and Evolutionary Rates

**FIGURE 5:**
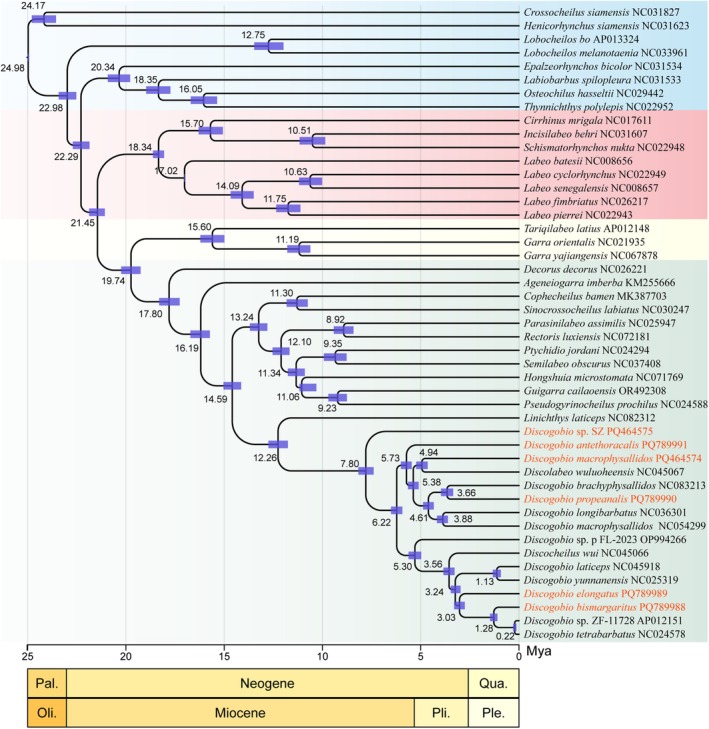
Divergence time estimation inferred via Bayesian relaxed dating methods (BEAST) based on the nucleotide sequences of 13 PCGs. Node bars indicate the 95% confidence intervals of the divergence time estimate.

Our current study determined that the divergence time to the most recent common ancestor (TMRCA) of the genera *Discogobio* and *Discolabeo* was approximately 7.80 million years ago, at the end of the Miocene period (Figure 5). We postulate that the geological upheavals and climatic conditions during the middle Tertiary might have triggered the divergence of *Discogobio*. The collision between the Indian Plate of the former southern continent and the ancient Asian Plate during the Late Eocene, followed by its northward subduction, led to the uplift of the Qinghai‐Xizang Plateau, forming what was known as the “Roof of the World” (Li et al. 2019). In the middle Tertiary, intense climatic and environmental fluctuations caused the plateau's surface to progressively flatten (Shi et al. 1999). This process saw the connection of numerous scattered lakes, giving rise to the rivers and the establishment of extensive river systems in and around the plateau, potentially creating favorable conditions for the dispersal of *Discogobio* (Jia et al. 2004; Zheng et al. 2005). However, our findings differ from those of previous studies (Wang et al. 2022; Che et al. 2024), which could potentially be ascribed to the broader taxonomic sampling of *Discogobio* species and the employment of distinct geological and fossil calibration points in our study.

**FIGURE 6:**
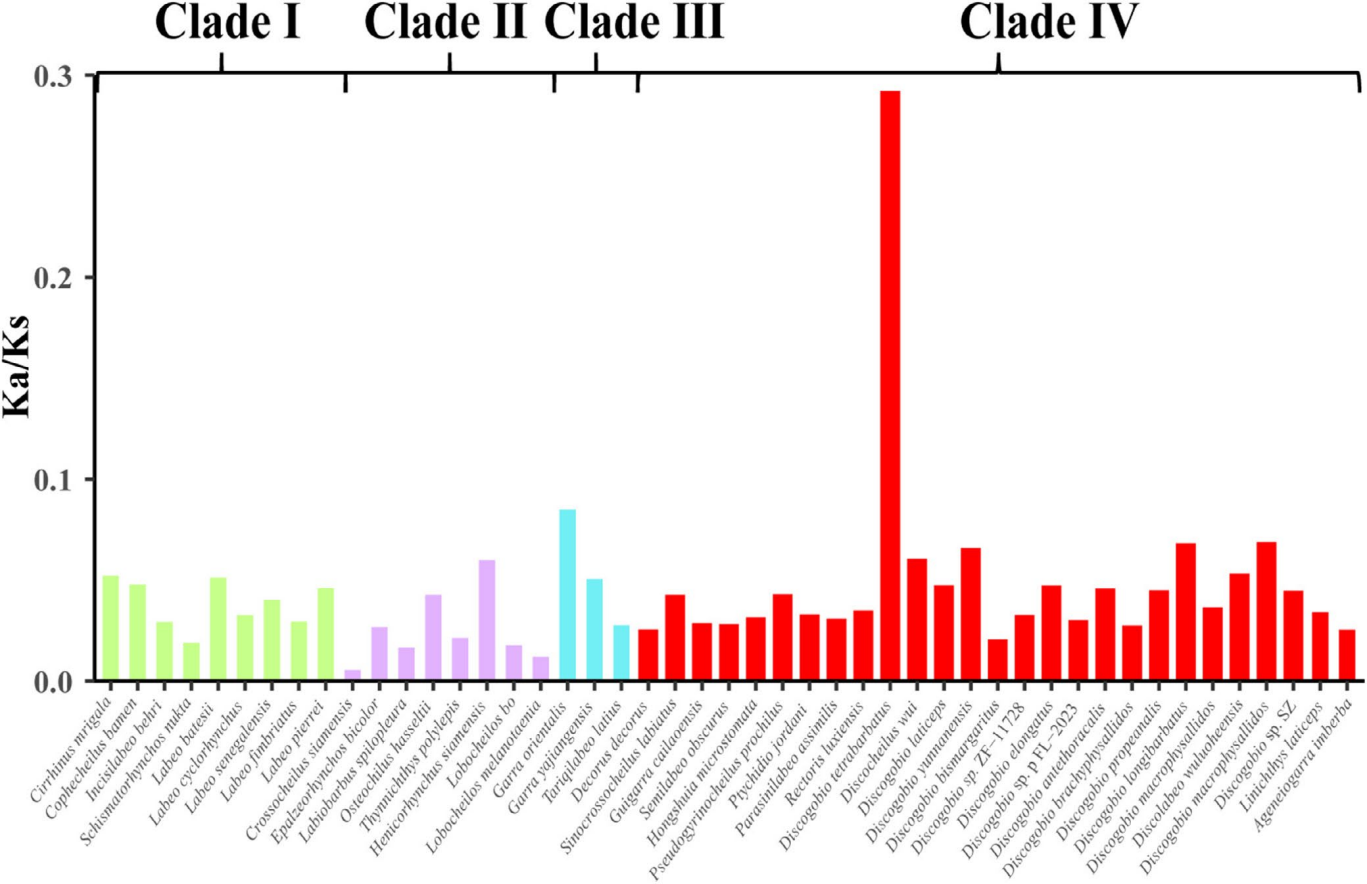
The evolutionary rates of the four major lineages based on the 13 concatenated PCGs.

Selection plays a crucial role in understanding how species adapt to their ecological environments (Yang et al. 2021). Birds, mammals, and fish living in cold climates have undergone strong purifying (negative) selection, evidenced by their Ka/Ks ratios being < 1 (Sun et al. 2011; Wang et al. 2021). The intense uplift of the Qinghai‐Xizang Plateau may change the climate or environment of this area, causing many species to undergo adaptive evolution, such as schizothoracine fish (He and Chen 2007) and *Triplophysa* fish (Wang et al. 2016). In this study, we analyzed the evolutionary rates of the PCGs to derive the independent Ka/Ks ratios for 14 *Discogobio* species and 33 other Labeoninae species (Figure 6). As shown in Figure 6, the Ka/Ks ratios for *Discogobio* species ranged from a maximum of 0.2912 for *D. tetrabarbatus* to a minimum of 0.0197 for DB. The analysis indicated that all Ka/Ks ratios were < 1, suggesting that all species in our study have undergone purifying selection. Briefly, the overall Ka/Ks ratios suggested that *Discogobio* species may experience stronger purifying selection to eliminate harmful mutations and adapt to the changing environment (Yang et al. 2021).

## 4 Conclusion

In this study, we obtained mitogenome sequences from six *Discogobio* species by conventional overlapping PCR and Sanger sequencing. Each mitogenome consisted of 13 PCGs, 22 tRNAs, two rRNAs, and one single control region. The genome size, gene order, and nucleotide composition of these six mitogenomes were similar to those of previously reported species of the subfamily Labeoninae. Most PCGs began with an ATG codon and ended with TAA, TA, or T codons. The low Ka/Ks ratios indicated that PCGs in these *Discogobio* species were under purifying selection. The subfamily Labeoninae was composed of four major clades. Additionally, the phylogenetic trees strongly supported the non‐monophyly of *Discogobio*. The most recent common ancestor of *Discogobio* was estimated to have existed approximately 7.80 Mya. This study provided information on the mitogenome characteristics, phylogenetic relationships, and adaptive evolution of *Discogobio*, as well as genetic data for the conservation of *Discogobio*.

## Author Contributions

**Huan Cheng:** conceptualization (equal), data curation (lead), formal analysis (equal), investigation (equal), methodology (equal), software (lead), visualization (lead), writing – original draft (lead), writing – review and editing (equal). **Renrong Huang:** investigation (equal), methodology (equal), visualization (supporting), writing – review and editing (supporting). **Hongmei Li:** formal analysis (supporting), investigation (equal), resources (equal), writing – review and editing (supporting). **Zhenya Qiu:** methodology (supporting). **Shan Xiong:** methodology (supporting). **Renyi Zhang:** conceptualization (lead), data curation (equal), formal analysis (equal), funding acquisition (lead), investigation (equal), methodology (supporting), resources (lead), software (supporting), visualization (supporting), writing – review and editing (lead).

## Ethics Statement

In this research, *Discogobio* fish samples were collected from public areas that did not require specific permits because the species is neither endangered nor protected. The samples (dead individuals) were obtained through donations from Hongfu Yang and purchases from local fishermen.

## Conflicts of Interest

The authors declare no conflicts of interest.

## Supporting information


**Figure S1.** Relative synonymous codon usage (RSCU) in the mitogenomes of six species. The values shown on the chart indicate the distribution percentage of each amino acid across the different species.

**Figure S2.** Predicted secondary cloverleaf structures for the 22 transfer RNA genes of *D. antethoracalis*.

**Figure S3.** Predicted secondary cloverleaf structures for the 22 transfer RNA genes of *Discogobio* sp. SZ.

**Figure S4.** Predicted secondary cloverleaf structures for the 22 transfer RNA genes of *D. macrophysallidos* .

**Figure S5.** Predicted secondary cloverleaf structures for the 22 transfer RNA genes of *D. propeanalis*.

**Figure S6.** Predicted secondary cloverleaf structures for the 22 transfer RNA genes of *D. bismargaritus* .

**Figure S7.** Predicted secondary cloverleaf structures for the 22 transfer RNA genes of *D. elongatus* .

**Table S1.** Primers used for PCR in this study.

Table S2.

Table S3.

Table S4.

## Data Availability

The complete mitogenomes of six *Discogobio* species were deposited in the GenBank of NCBI under accession numbers PQ464574–PQ464575 and PQ789988–PQ789991.
