# Supplementary figures and images for: Mitochondrial Genomes of Six Discogobio Species (Teleostei, Cyprinidae) and Their Phylogenetic Analysis
Source: Ecol Evol. 2025 Mar 17;15(3):e71142. doi: 10.1002/ece3.71142 (PMC11917117; doi:10.1002/ece3.71142)

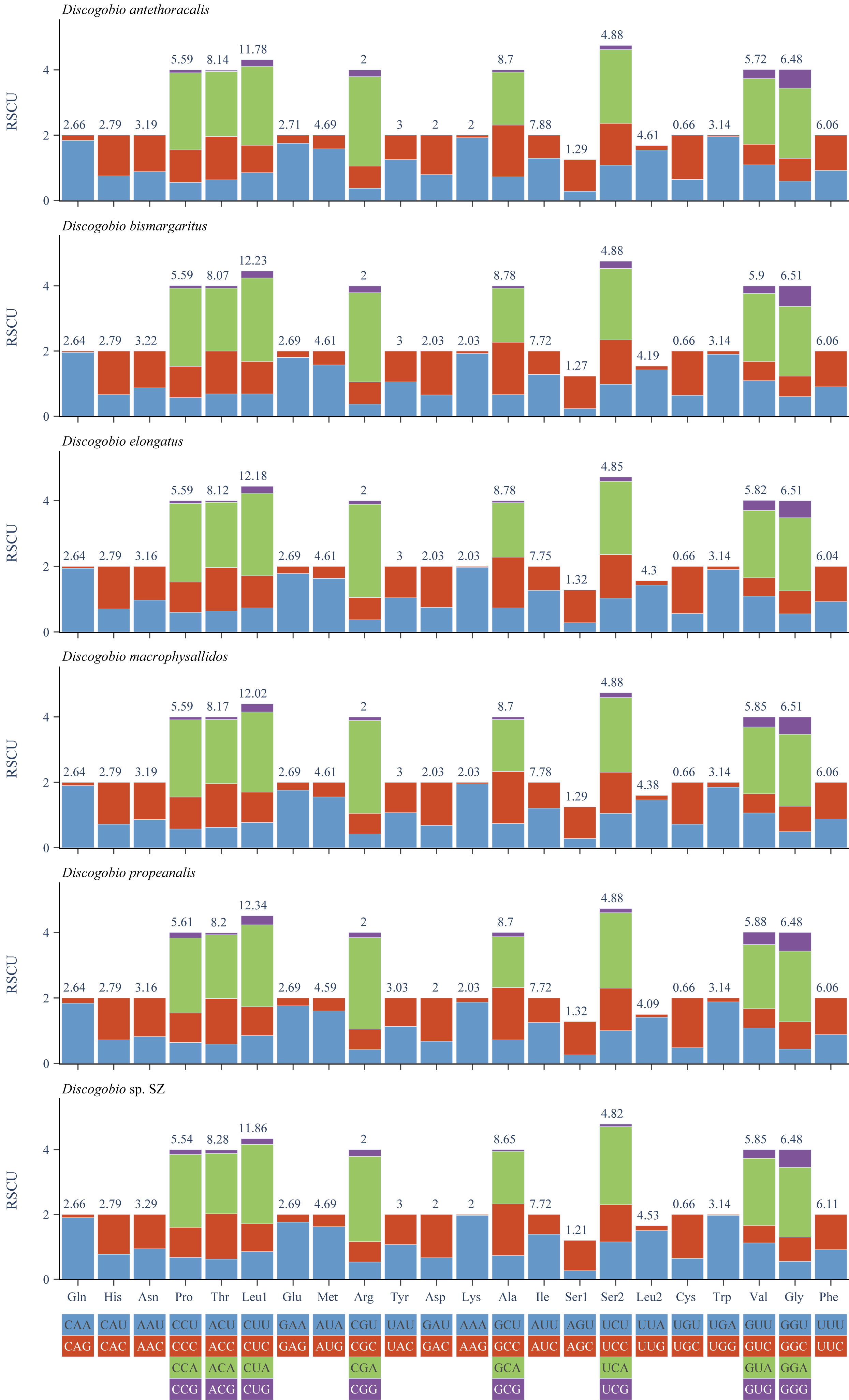

Supplement: Supplementary file 1 — Figure S1. Relative synonymous codon usage (RSCU) in the mitogenomes of six species. The values shown on the chart indicate the distribution percentage of each amino acid across the different species. [file ECE3-15-e71142-s011.tif]

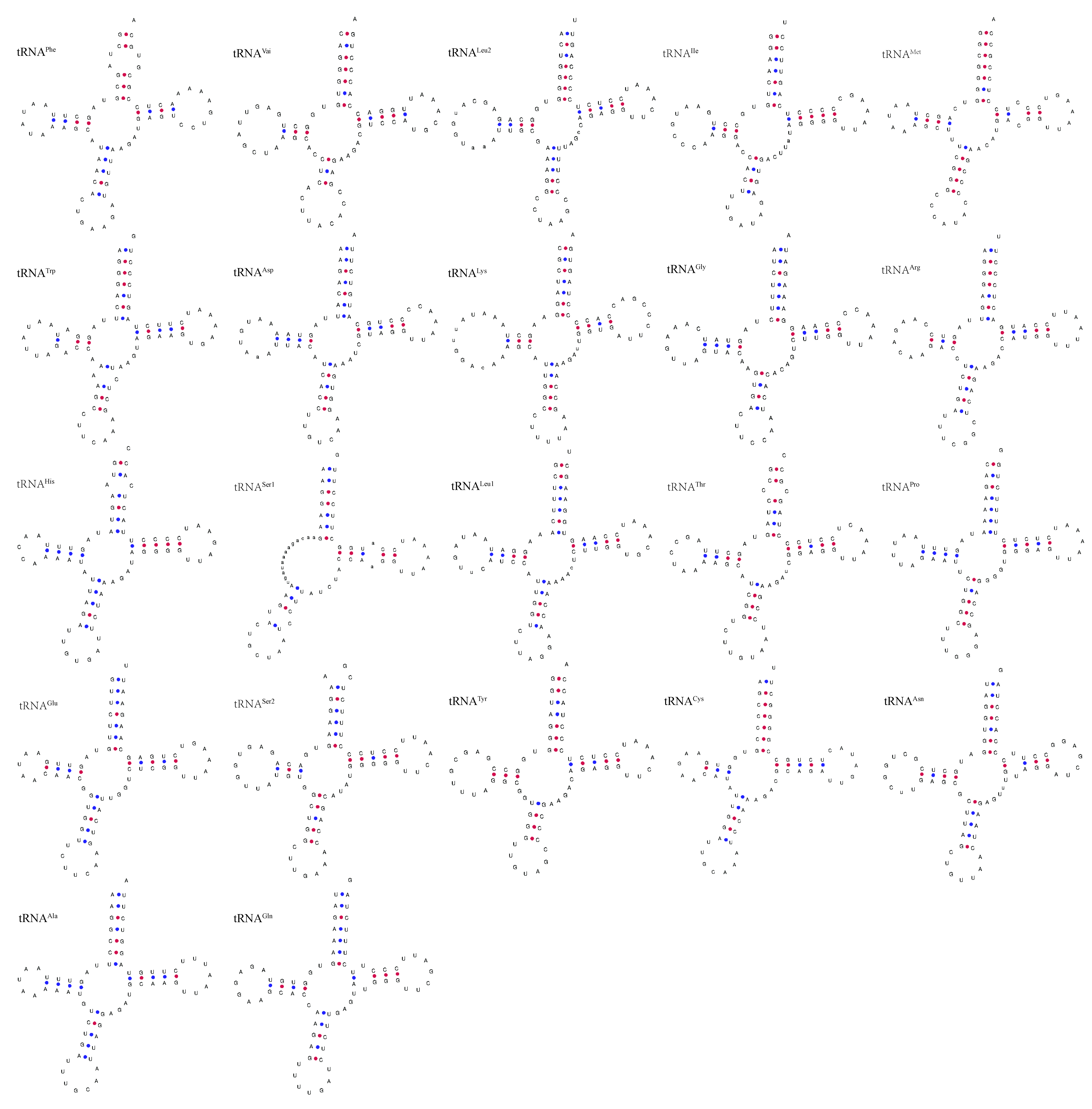

Supplement: Supplementary file 4 — Figure S4. Predicted secondary cloverleaf structures for the 22 transfer RNA genes of D. macrophysallidos . [file ECE3-15-e71142-s001.tif]
