# Supplementary material for: Mitochondrial Genomes of Six Discogobio Species (Teleostei, Cyprinidae) and Their Phylogenetic Analysis
Source: Ecol Evol. 2025 Mar 17;15(3):e71142. doi: 10.1002/ece3.71142 (PMC11917117; doi:10.1002/ece3.71142)
Supplement: Supplementary file 8 — Table S1. Primers used for PCR in this study. [file ECE3-15-e71142-s008.docx]

**Table S1** Primers used for PCR in this study.

| **Primer name** | **Primer sequence** | **Annealing T (**°C**)** |
| --- | --- | --- |
| PF1 | 5′- GCACCTCACTTACACCGA-3′ | 46°C |
| PR1 | 5′- TGAACAAACGAACCCTTA-3′ |  |
| PF2 | 5′- CGACCACGGAGGAAAAAA-3′ | 55°C |
| PR2 | 5′- CTTGGGCATTCAGGCACA-3′ |  |
| PF3 | 5′- CCCTTCGCCCTATTTTTCC-3′ | 45°C |
| PR3 | 5′- GGGGGGTCTGGGTTGTCT-3′ |  |
| PF4 | 5′-CAATACGCCCCCCAACTAA-3′ | 49°C |
| PR4 | 5′-GTAATACCAGCGGCTAAAACG-3′ |  |
| PF5 | 5′-CATCATTCCTGCTTCTCCT-3′ | 46°C |
| PR5 | 5′-TAACCTACGATTTCACCTTG-3′ |  |
| PF6 | 5′-CAAACTAACGAGAAAGGGA-3′ | 46°C |
| PR6 | 5′-TGGTGTAAAGGTGTATGGAA-3′ |  |
| PF7 | 5′-CACCAAATGAACCAACCC-3′ | 46°C |
| PR7 | 5′-CGTAAACTCCATCTGCGA-3′ |  |
| PF8 | 5′- GCACCTCACTTACACCGA-3′ | 56°C |
| PR8 | 5′- TAGAGGGGTTGATAATGGGT-3′ |  |
| PF9 | 5′-GCTTTCTCCGCTTGTGAA-3′ | 52°C |
| PR9 | 5′-CGAGTGTGGAATGGTTGG-3′ |  |
| PF10 | 5′-CGAACACACAGCCGAACA-3′ | 49°C |
| PR10 | 5′-GCTCCTAAGCAGAGGCAAAT-3′ |  |
| PF11 | 5′-GATGACACGGACGAGCAG-3′ | 48°C |
| PR11 | 5′- AGGGTTAGGAGGAAAATGG-3′ |  |
| PF12 | 5′-CCAACCATAACAATACCAAC-3′ | 46°C |
| PR12 | 5′-AGTAATAAAAGGACAACGCC-3′ |  |
| PF13 | 5′-AACATACACGCAAACGGA-3′ | 46°C |
| PR13 | 5′-TGCCAAGTGAAAAGAAACC-3′ |  |
| PF14 | 5′-ATTACTGGCATCTGGTTCC-3′ | 57°C |
| PR14 | 5′-CCATTTGGCTTTATTTTTG-3′ |  |
| PF15 | 5′-TGAGTATGCCCTCAATCC-3′ | 48°C |
| PR15 | 5′-GCAACCAGCTATCACCAG-3′ |  |
